# Unprofessional behaviour of GP residents leading to a dismissal dispute: characteristics and outcomes of those who appeal

**DOI:** 10.1186/s12875-024-02294-8

**Published:** 2024-02-20

**Authors:** Judith A. Godschalx-Dekker, Charlotte A. M. Sijbom, Pieter C. Barnhoorn, Walther N. K. A. van Mook

**Affiliations:** 1 Department of Psychiatry and Medical Psychology Flevoziekenhuis, Almere, The Netherlands; 2 GGZ Central Flevoland, Almere, The Netherlands; 3 Department of Occupational Medicine, HumanCapitalCare, Son, The Netherlands; 4 Department of Public Health and Primary Care, Leiden University Medical Center, Leiden, The Netherlands; 5 Department of Intensive Care Medicine, Maastricht UMC+, Maastricht, The Netherlands; 6 Academy for Postgraduate Training, Maastricht UMC+, Maastricht, The Netherlands; 7 School of Health Professions Education, Maastricht University, Maastricht, The Netherlands

**Keywords:** Professionalism lapses, Unprofessional behaviour, Professionalism, Professional identity formation (PIF), General Practice (GP), Residents, Internship and residency, Remediation, Postgraduate Medical Education (PGME), Continuing Medical Education (CME).

## Abstract

**Background:**

Recognition of poor performance in General Practice trainees is important because underperformance compromises patients’ health and safety. However, in General Practice, research on persistent underperformance while in training and its ultimate consequences is almost completely lacking. We aim to explore the unprofessional behaviours of residents in General Practice who were dismissed from training and who litigated against dismissal.

**Methods:**

We performed a structured analysis using open-source data from all General Practice cases before the Conciliation Board of the Royal Dutch Medical Association between 2011 and 2020. Anonymised law cases about residents from all Dutch GP training programmes were analysed in terms of the quantitative and qualitative aspects related to performance.

**Results:**

Between 2011 and 2020, 24 residents who were dismissed from training challenged their programme director’s decision. Dismissed residents performed poorly in several competencies, including communication, medical expertise and most prominently, professionalism. Over 90% of dismissed residents failed on professionalism. Most lacked self-awareness and/or failed to profit from feedback. Approximately 80% failed on communication, and about 60% on medical expertise as well. A large majority (more than 80%) of dismissed residents had previously participated in some form of remediation.

**Conclusions:**

Deficiencies in both professionalism and communication were the most prevalent findings among the dismissed General Practice residents. These two deficiencies overlapped considerably. Dismissed residents who challenged their programme director’s decision were considered to lack self-awareness, which requires introspection and the appreciation of feedback from others.

**Supplementary Information:**

The online version contains supplementary material available at 10.1186/s12875-024-02294-8.

## Background

General Practitioners prepare residents for all facets of future practice, including professional behaviour. Assessing professional behaviour in residency training received more attention in the Netherlands after competence-based education was introduced into the residency training programmes in general, and General Practice (GP) specifically in 2006 [1, 2]. 

Nevertheless, assessing professional behaviour among GP residents remains a difficult task for staff delivering training [3], in part because of a lack of cultural consensus, and the resultant breadth of the definition of “high personal standards of behaviour” [4]. Despite this difficulty, assessing professionalism during residency is important because lapses in professional behaviour form a continuum from medical school to clinical practice [5, 6], and are related to patient complaints [7] in both underperforming GPs and residents [8, 9]. For patients, unprofessional behaviour of doctors (in training) might result in sub-optimal outcomes regarding patients’ health, safety, and care, undermining patients’ perceptions of the trustworthiness of the individual physician as well as the profession as a whole. For colleagues, such behaviours demoralise and compromise collaboration as well as negatively impact the well-being of staff [10, 11]. Unprofessional behaviour in residency might have consequences for continuation of training. If residents do not sufficiently improve their professional behaviour, their programme director may ultimately decide to dismiss them. Dismissal is thus the end-stage of a process of unsuccessful remediation attempts. In the Netherlands, 9 to 23% of the GP residents *temporarily* underperform, requiring extra attention or remedial measures from their training staff [12, 13]. Conversely, *patterns* of unprofessional behaviour are difficult to remediate and commonly recognized in the last part of residency training [13]. In general, programme directors experience the dismissal process as difficult and complicated [14]. 

Reasons for dismissal, in both undergraduate medical education [15] and postgraduate training programmes [16–18], might ultimately consist of unprofessional behaviour related to underperformance in several other competencies. Three studies of poorly-performing GP residents suggested that lapses in professionalism, communication, and medical expertise were the most common problems [12, 13, 19]. In these studies, the dismissed GP residents were small in number, and their unprofessional behaviours were not explicitly described. Qualifying unprofessional behaviour enables practitioners to address poor performance in practice specifically.

This study aimed to classify and describe the characteristics of GP residents in the Netherlands who litigated before the Dutch National Conciliation Board against dismissal from their GP training. More specifically, our research focused on identifying deficiencies in the CanMEDS competency domains [4], the extent to which they overlapped within an individual, and which typical qualifications the programme director used to describe the unprofessional behaviour.

## Methods

### Data collection

We performed a retrospective case study of all conciliations registered at the Royal Dutch Medical Association of GP residents dismissed between 2011 and 2020.

### Context and setting: postgraduate GP training

In their first and last years, residents in Dutch postgraduate GP training are supervised by an experienced GP. Once weekly, small groups of residents are trained in theoretical, practical and reflective skills. Three monthly, performance in a variety of competencies is evaluated according to the National Assessment Protocol. This protocol includes videotaped assessments of physician-patient contacts. Supplementary Appendix 1 displays the competency assessment list (Compass) for professionalism (responsibility, self-care, self-directed learning, reflection, ethics, respect) [20]. Biannually, the residents’ knowledge is formatively tested using the National GP Knowledge Test. At the end of each year, the programme director, with the input of the residents’ teachers and clinical supervisors, decides whether they can continue training, with or without additional conditions. If the resident fails to meet the requirements, the programme director may decide to dismiss them. After dismissal, the resident may first request mediation, and second conciliation, from the board of the Royal Dutch Medical Association (RGS KNMG)(see below).

### Conciliation by the royal dutch medical association

The Registration Committee for medical Specialists (RGS) from Royal Dutch Medical Association (KNMG) periodically checks whether physicians and training programmes (including General Practice) comply with the quality criteria of the College Medical Specialists. One of the RGS’ boards is the Conciliation Board, a national board consisting of two legal professionals (a chairman and a clerk), a programme director and a resident. The Conciliation Board evaluates whether the programme director has made a deliberate and careful decision and followed due process. The board organises a hearing in which the resident and the programme director explain their position on the disputed decision. The resident may contest the programme director’s assessment, the consistency of the applied assessment protocol, and/or the guidance and remediation that was offered. The programme director explains why the resident is considered unsuitable for General Practice and why further remediation is considered not to result in sufficient improvement. Several weeks after the hearing a written decision is sent to the resident and programme director. All anonymised decisions of the Conciliation Board between 2011 and 2021 are publicly available online [21]. Since 2021, only summaries have been published.

### Data analysis

The principal investigator (J.G.) selected the anonymised decisions based on specialty (General Practice), type of dispute (dismissal of residency) and litigants (programme director versus resident), collected the residents’ general characteristics and calculated descriptive statistics (% or mean) using Microsoft Excel (version 2202). The principal investigator then scored each case in terms of deficiencies in competency domains. In almost all cases the programme director’s argumentation specifically mentioned the domains of the residents’ insufficiencies. In cases where a decision did not specify in which CanMEDS competency domain(s) the resident was considered deficient, the principal investigator reasoned in which domain the reported deficiencies would fit (e.g., lack of conversation skills would fit the domain communication). The descriptions of unprofessional behaviours were copied verbatim from the board’s decisions. These descriptions were classified using the definitions of unprofessional behaviour in medical students developed by Mak-Van der Vossen et al. [22], and in line with focus groups among GP training staff by Barnhoorn et al. 2021 [23]. 

## Results

Between 2011 and 2020, 24 dismissed GP residents challenged their programme director’s decision. Table 1 shows their characteristics. Gender was equally distributed. About 30% of the dismissed residents were on sick leave. Health issues reported in those on sick leave were hospital admission, contusion cerebri, attention deficit hyperactivity disorder, neurological disorder, and nervous exhaustion complaints. Insufficient Dutch language skills were present in approximately 30% of dismissed residents. These insufficient language skills manifested as problems with understanding, pronunciation, and vocabulary. Most residents (83%) participated in some form of remediation prior to the dismissal, such as coaching and mentoring, resits of assessments or rotations, speech or languages courses, or reducing their workload by working part-time. Remediation was not offered or applied to all residents, e.g. because of sick leave and in a case of severe fraudulent behaviour, or the programme director considered further remediation unproductive. This was either because the remaining time in the training programme was too short to result in sufficient improvement or because the resident was considered to have had enough prior chances and opportunities to change without the desired results.

**Table 1:** Characteristics and aspects of poor performance

	**Individual characteristics (** ***n*** ** = 24)**	
	**%**	***(n)***
Gender, % male	50.0	(12)
Sick leave during training	33.3	(8)
Previously enrolled as a GP resident	12.5	(3)
Insufficient on patient communication test	20.8	(5)
Linguistic or cultural issues	33.3	(8)
Insufficient on at least one knowledge test	70.8	(17)
Attended a remediation programme	83.3	(20)
	**Training characteristics (** ***n*** ** = 24)**	
	**Mean**	**Range**
Years of training until detecting first problems	1.0	0–2
Years of training until dismissal	2.1	0.25-3
Number of insufficient competencies	3.0	0–7
Number of themes of unprofessional behaviour	1.5	0–3
	**Insufficient competencies (** ***n*** ** = 23)**	
	**%**	***(n)***
Professionalism	95.7	(22)
Communication	82.6	(19)
Medical Expertise	58.3	(14)
Management	39.1	(9)
Collaboration	21.7	(5)
Scholarship	13.0	(3)
Health Advocacy	4.3	(1)

### Competencies

Most residents, performed poorly in professionalism (96%), and communication (83%), while a smaller number performed poorly in medical expertise (58%). All residents who performed poorly on communication also underperformed on professionalism. On average, dismissed residents performed poorly on three competencies. Of the 24 dismissed residents, 22 performed insufficiently on professionalism. Of the two, one resident was dismissed from training because persistent illness left too little time remaining in the training programme to assess competencies, and the other resident was dismissed exclusively due to insufficient medical expertise.

### Unprofessional behaviour

The unprofessional behaviour of dismissed residents showed considerable overlap between the four types of unprofessional behaviour (failure to engage, dishonesty or disrespect, poor self-awareness) as previously described by Mak-van der Vossen et al. [22, 23] Table 2 shows the quantification and qualification of unprofessional behaviours displayed by these residents. We discuss below, the four types of unprofessional behaviour, including a subcategorization of poor self-awareness (follow-up after feedback, superficial self-reflection, personal circumstances), as well as examples of the language used by programme directors to describe problematic behavioural patterns. The programme directors outlined a pattern of behaviours that requires attention from the resident and staff and the suggested advice or guidance for the resident’s improvement. In the available documentation, some situations are described comprehensively, whereas others contain little detail, with loose statements or opinions isolated from context.

**Table 2:** Aspects of insufficient professionalism (*n* = 22)

Categories*	% (*n*)	Examples
Failure to engage	40.9 (9)	Absent on shifts or education days
		No active learning attitude, including not participating in education or insufficient upkeep of portfolio
		Avoiding providing care for specific patient groups
Dishonest behaviour	9.1 (2)	Resumé fraud
		Incorrect registration of working hours
Disrespectful behaviour	27.3 (6)	Conflicts with staff
		Patient complaints
		Lack of empathy
		Cynicism
Poor self-awareness	86.4 (19)	Insufficient follow-up after feedback
		Avoiding or externalizing feedback
		Inability to take responsibility for their personal share in insufficiencies
		Inability to accept or profit from feedback
		Inability to adjust behaviour
		Superficial self-reflection
		Insufficient reflection on actions/situations/consultations
		Lack of self-reflection or introspection
		Lack of insight into competence limitations, and acting beyond level of competence

*Adapted from Mak-Van der Vossen et al. 2017.

#### Failure to engage

Residents must make it apparent to their clinical supervisor that they take responsibility for the agreements made [24]. In 41% (9/22) of the cases lacking professionalism, the resident failed to sufficiently engage in the learning process or in patient care. Residents lacking engagement, for example, were those who did not adhere to agreements, missed appointments without giving prior notice or who, without giving notice, failed to show up for shifts on the emergency ward or education days. In one case, teachers deemed a resident to have insufficient professionalism because a proactive attitude towards self-directed learning and a desire to strive for excellence and improvement were lacking [25]. Lack of engagement by residents was apparent in cases where they avoided caring for specific patient groups such as: elderly patients in a nursing home; psychiatric patients; palliative patients; or patients in clinical surgery. Most residents lacking engagement (7/9) were also considered to lack respectful behaviour, self-awareness, or both.

#### Dishonest behaviour

Two residents (of 22; 9%) were dismissed for dishonesty in that they committed fraud. One resident was accused of (and admitted) resumé-fraud. The resident lied about a previous failed participation in another GP-training programme, which was discovered in the first months of their residency [26]. The other resident incorrectly registered too many working hours [27]. 

#### Disrespectful behaviour

In 27% (6/22) of the cases lacking professionalism, residents were disrespectful towards patients or staff. Disrespect was displayed during conflicts with, among others, medical specialists, assistants, or clinical supervisors, and led to premature changes of supervisors or rotations [28–30]. Where reported, disrespectful behaviour was present in combination with other unprofessional behaviours. An example of this is case [31] in which lack of respect, self-awareness and engagement in interaction with supervisors, peers, and patients were all reported. According to the supervisors, this resident lacked empathy, emotional awareness, and involvement during patient consultations. He handled differences of opinion unconstructively and inconsistently, and because of his critical attitude, he could come across as a ‘know-it-all’.

#### Lack of self-awareness

Most dismissed residents (19/22) lacked self-awareness. In addition, more than half (11/19) were lacking respect or engagement. In the next section, we discuss case examples highlighting the language used by programme directors to address problematic behavioural patterns resulting from a lack of self-awareness. These patterns were related to feedback, reflection, and personal circumstances, including cross-cultural challenges.

#### Follow-up after feedback

Training staff regarded dealing constructively with feedback as an indispensable quality. Examples of the attitude residents should have had in response to feedback received in residency training include.

- Feedback should not feel like an assault, and the resident should not respond reactively, but proactively [24].
- Residents should open themselves up to reactions from peers, and need to respond less defensively to feedback. They needed to consider the influence of their “personality, personal and professional past with a little more distance” [31].
- Residents should keep their teachers up to speed about the learning process. In some cases, teachers noticed that “little action was taken on feedback, or if it was, there was no communication about it” [32].
- Residents should not continue to “follow their own path” in terms of communication and feedback, failing to recognize or accept the feedback received from those involved in their training [33].

#### Superficial self-reflection

Programme directors, clinical supervisors and teachers regularly reported a lack of reflection or self-reflection among dismissed residents. We provide a few examples of how they referred to shortcomings in residents’ reflection, and/or how they should be addressed. Some of these shortcomings are also related to other facets of professionalism, such as dealing with feedback, lack of involvement and interaction.

Reflection was seen as a “skill”, a “capacity” or a “competency” that could be learned in residency training, with specific attention and/or extension of training [34]. In one case, a resident needed help with reflection on their own functioning because this would improve their chances success in residency training: “In a conversation with the programme director the resident had to reflect on two recent and ten previously written letters, and the progress made during the current rotation.” However, “no growth in reflective competencies” was observed. The resident showed no insight into the background of bottlenecks that had been signalled and great concern was expressed about their “teachability and self-reflection” [35]. In general, residents should reflect on “events/measures/tools” [24] and apply “consultation transcending reflection” [33]. 

According to teachers, a resident should reflect on their actions and adopt an accountable attitude. One of the residents, however, previously left their reflection group [31]. Another should “continue to work on self-reflection and being critical” of themself [34]. In another case, the “depth of reflection” was judged as “superficial”. This resident was not “open to discussing knowledge gaps”, moreover, they were unable to ask for help, which might contribute to unsolved problems or dangerous situations for patients [36]. 

#### Personal circumstances

One programme director stated that the way a resident dealt with personal problems was unprofessional: “the resident has been insufficiently present and accountable” [32]. The teacher of another thought that a resident was not up to the “responsibilities of the profession as a general practitioner”, due to concerns about “self-care in relation to the profession of general practitioner”. In addition, this resident was considered to have “insufficient insight into their own performance” [24]. In one case, a resident felt they had been exposed to cultural discrimination and pointed out the need for a supervisor who could provide confidence and space, with respect for the resident’s learning style [36]. Another resident was mandated to discuss with their mentor which aspects of their cultural background could potentially hold back their improvement [33]. The cultural aspects referred to were not specifically described.

## Discussion

### Statement of principal findings

Between 2011 and 2020, 24 residents dismissed from GP residency training challenged their programme director’s decision. On average, these residents were considered to be underperforming in three competencies, namely professionalism, communication, and medical expertise. There was considerable overlap between these domains. Most residents who were considered unprofessional and challenged the programme director’s decision to dismiss, lacked sufficient levels of self-awareness. The language programme directors used to describe the reasons underlying dismissal due of failure of engagement, included terms such as “not adhering to agreements or appointments”, “avoiding provision of care to specific patient groups”, or “the lack of a proactive attitude towards self-directed learning”. Dishonest residents’ behaviour was mainly fraud, whereas disrespectful behaviour was mainly related to coming in conflict with others. Residents with poor-self-awareness were unable to reflect on events, consultations and their performance, lacking growth or the development of reflective competencies. These residents were unable to accept feedback and communicate how they were able to apply and learn from it (lacking self-awareness).

### Findings in relation to previous research

The Conciliation Board upheld the decision of the programme director in 84% of cases [37]. Half of the dismissed GP residents were male, while most GP residents in training were female (76% from 2010 to 2018) [38]. This finding is in line with the literature on probation in which males are overrepresented [12, 39]. Language difficulties were present in approximately 33% of the dismissed residents in the present study. The fact that only 11% of the Dutch-born higher educated population does not speak Dutch as their mother tongue [40], could, to some extent, be contributory to male residents of foreign descent being dismissed disproportionately frequently, despite a, for example, selection process that includes assessment of language skills. Twelve and a half percent of dismissed residents in the present study had previously been in another GP-residency training program. In some cases, this was several years ago, suggesting that these residents were older than their peers, and/or had had multiple training attempts. Our findings on language, gender, and presumed older age of residents are in line with the general literature on GP-residents with poor performance, residents on probation, and medical students who are summoned before a professional behaviour board [11, 12, 39]. In the present study around 30% of the dismissed residents were on sick leave, whereas Vermeulen [13] found that during the course of their residency programmes, only 14% of Dutch GP residents went on sick leave. Possibly residents with sick leave, sickness, or disabilities are less likely to graduate. Such an association was indeed found in undergraduate medical students with disabilities [41], and psychiatry residents [42]. Reasons for sick leave, however, may be diverse. Illness may be a source of deficiencies, preventing optimal performance, or may even be the result of the stresses associated with a remediation trajectory. Sick leave might indicate that the resident cannot perform, perhaps due to psychological factors, such as shortness of resilience, being overwhelmed with work-related experiences, exhaustion from struggling to keep up, and avoiding assessment, which could potentially yield negative feedback.

The findings of our study, that most shortcomings of poorly performing dismissed residents fell into the competency domains of professionalism, communication, or medical expertise, are in line with the findings of Vermeulen et al. [13], who studied the GP resident portfolios of 215 trainees from a single Dutch university hospital. They found that temporarily poor performance was common, most frequently in the areas of communication (29%), medical expertise (27%), and professionalism (23%). However, residents deficient in professionalism were, on average, more often deficient in multiple competencies when compared to other residents with poor performance. This is in line with the significant overlap of deficiencies in distinct competencies identified in the present study.

The results of the present study also partly align with the findings of Van Moppes et al. [12], who quantitatively studied the educational success of 1700 GP residents who started their residency at one of the seven Dutch university hospitals in 2015, 2016, or 2017. Here, 9% (154) of these residents had an underperformance event, such as having to follow a mandatory coaching pathway (86%; 133/154). These underperformance events were related to communication (57%; 88/154), organisation, collaboration or health advocacy (66%; 101/154), medical expertise or scholarship (51%; 79/154), or professional integrity (45%; 70/154). In contrast to the present findings, professionalism, which was described as: balancing personal and professional roles, and working consistently on improving professional skills, was their least-mentioned deficiency. Their definition of professionalism lacks aspects dealing ‘consciously with differences in norms and values’ as referred to in the competence assessment list of professionalism (Compass) from the 2019 GP National Assessment Protocol (see Supplementary Appendix 1) [20]. Consequently, the moral development of the resident, recognition of someone’s boundaries of competency and care, and interaction with respect were possibly not assessed. The present study shows that the Dutch programme directors’ view of (insufficient) professional behaviour, is in fact more broad and extensive, and takes into account patterns of disrespectful interaction, dishonesty, disengagement or unawareness of self, and inability to reflect. The finding that lack of self-awareness was the performance issue most commonly mentioned by programme directors, is compatible with the categorization of Mak-Van der Vossen et al. of unprofessional behaviour in medical school [22]. ‘Lack of self-awareness’ and ‘superficial self-reflection’ impact performance on other competencies. For example, insufficient insight into performance might compromise medical expertise. These deficiencies of self-awareness may occur more frequently in residents who dispute their dismissal.

In the present study, 91% of the dismissed residents underperforming in the area of professional behaviour failed remediation. Underperforming residents previously studied had lower percentages of unprofessional behaviour, however most residents in those studies successfully completed their remediation and consequently completed their training [12, 13]. Furthermore, the definition and categorisation of unprofessional behaviour may vary in various studies. The present study’s findings are nevertheless in line with the literature since problems in professionalism are difficult to remediate, especially when related to personality structure, convictions, and values [19]. 

### Strengths and limitations

This study has several strengths. One is its originality, as it is one of the few that gives insight into the reasons for GP residents dismissal from a data source which provides extensive and detailed expressions of programme directors’ motives. Other strengths include the completeness, open access, and uniqueness of the data source. While most appeal procedures are confidential [43], the anonymised decisions analysed in this study are publicly available and can be used to verify our results [20]. Moreover, the present source material stems from actual legal decisions from external assessors, highlighting the reliability of the data over programme director surveys. The Dutch GP specialty training is comparable with most European, British and US GP specialty training programmes in terms of similar characteristics and values, such as a solid academic basis, a competence-based approach, and longitudinal assessments [12]. 

However, there are also methodological limitations. The results reflect the judgments of the programme directors involved in disputes, and the arguments they used before the board. Thus, the cases are with respect to disputed decisions, and especially lack information that was not part of the case. Information about dismissed residents who neither appealed nor withdrew from the procedure, remains confidential as well, and is unfortunately inaccessible for comparison. We estimated the population of residents dismissed without dispute at about two-thirds of the residents dismissed (calculated with the drop-outs prompted by educational institutes from Van Moppes nationwide Dutch residency cohort of 23 drop-outs who started training between 2015 and 2017) [12]. However, the analysis of accessible law cases provides illustrative examples for training staff to identify and address unprofessional behaviour from residents who are unable to recognise their shortcomings and has the potential to improve the sensitivity of GP training staff to detect residents who require remediation.

### Implications and suggestions for further studies

This study highlights the need for further research with a more detailed focus on factors related to dismissal decisions to improve the assessment, teaching, and remediation of GP training candidates and GP residents. The relevance of acquiring self-awareness enabling the resident to regulate their behaviour and improve their performance through remediation is well accepted [22, 44–46]. However, how to assess and remediate the level of self-awareness is still a puzzle, both in GP residents and GP trainees. Future research might seek to establish validated instruments to identify issues and remediation strategies concerning constructs such as self-awareness, self-reflection, self-monitoring, self-regulation, self-efficacy, and even self-leadership, and how these constructs prompt professional identity formation in postgraduate medical education, even though these are considered difficult topics to address [23, 47–49]. 

An interesting finding is the considerable number of dismissed residents who went on sick leave before dismissal. Further research is absolutely necessary to clarify the relationship between sick leave and professional performance problems in residents requiring remediation, since the interplay between illness and performance may be complex. Another intriguing finding inclining further research is the suggested overrepresentation of residents of foreign descent in cases of dismissed residents. Future research should explore factors concerning assessment bias, discrimination, and remediation strategies sensitive to language complexity and cultural diversity [50]. This study regarding unprofessional behaviour touches upon aspects, arguments, and attitudes among dismissed GP residents. However, this study and the current literature fail to elucidate specific underlying reasons for residents’ underperformance and/or the training staff’s motivations for removal or remediation. Therefore, studies regarding residents’ dismissal should be repeated in dept in other countries and include GP residents who accepted dismissal or dropped out of residency for different reasons. In addition, it may be helpful to review training programmes, and qualitatively study teachers’ and clinical supervisors’ perceptions of benefits or detriments regarding successful completion of residency.

## Conclusion

This unique study examining the reasons why programme directors dismiss GP residents who fail to meet the required competencies, revealed a high prevalence of deficiencies in professionalism and considerable overlap between deficiencies in professionalism and communication. Dismissed GP-residents who appealed and who were judged as unprofessional by their programme directors were commonly described as not adhering to agreements or appointments, avoiding patients care or lacking a proactive learning attitude (disengagement). Among dismissed residents, dishonest behaviour mainly comprised fraud, whereas disrespectful behaviour was proneness to conflict. Residents with poor-self-awareness were unable to reflect on events, consultations and/or their performance, lacking the ability to grow or develop reflective competencies based on feedback. We were able to provide specific examples of these types of behaviours, and have hopefully been able to contribute to the improvement of recognition, denomination, and remediation of residents’ (and future general partitioners’) behaviour.

### Electronic supplementary material

Below is the link to the electronic supplementary material.

Supplementary Material 1

## Data Availability

The data is publicly available online: https://www.knmg.nl/opleiding-herregistratie-carriere/rgs/wat-doet-de-rgs/bezwaar-beroep-en-geschil/geschillencommissie-geschillenprocedure/uitspraken-en-jaarverslagen-geschillencommissie.htm#Jaarverslagen_Geschillencommis_(Uitspraken_en_jaarverslagen_Ge)-anchor. Coded data is available from the corresponding author on reasonable request.
