# Supplementary material for: Unprofessional behaviour of GP residents leading to a dismissal dispute: characteristics and outcomes of those who appeal
Source: BMC Prim Care. 2024 Feb 20;25:61. doi: 10.1186/s12875-024-02294-8 (PMC10877848; doi:10.1186/s12875-024-02294-8)
Supplement: Supplementary file 1 — Supplementary Material 1 [file 12875_2024_2294_MOESM1_ESM.docx]

**Supplementary Appendix 1:** **Competency Assessment List (Compass) for professionalism in the 3rd year residency training for General Practice**

| The resident … | | | |
| --- | --- | --- | --- |
| 1… balances personal and professional roles. | | | |
|  | Responsibility |  | |
|  |  | … displays commitment and involvement, and adheres to promises, agreements and appointments. | |
|  |  | … accepts accountability for the consequences of their own actions, including after they have made mistakes. | |
|  |  | … is able to bear the responsibilities of the profession. | |
|  | Self-care |  |  |
|  |  | … balances the demands of the profession and the desire for a private life. | |
|  |  | … balances professional distance and empathic involvement towards the patient. | |
|  |  | … adequately copes with feelings of powerlessness or uncertainty during patient consultations and in groups. | |
| 2… works systematically and purposefully to improve professional performance. | | | |
|  | Self-directed learning | | |
|  |  | … works purposefully towards learning based on self-reflection, test results, and feedback, and evaluates learning results. | |
|  |  | … makes active use of the learning opportunities of the educational institute and provides their own contribution. | |
|  | Adaptation of reflection and feedback | | |
|  |  | … analyses own behaviour or professional performance in relation to others (patients, colleagues, teaching staff) and underlying motives. | |
|  |  | … is open to feedback from others and adjusts behaviour if necessary. | |
| 3… deals consciously with differences in norms and values between care providers and patients. | | | |
|  | Professional ethics | | |
|  |  | … recognises boundaries of medical care and concurrent dilemmas, expectations and desires of others, including patients and colleagues. | |
|  |  | … handles moral questions and dilemmas with care. | |
|  | Respect |  | |
|  |  | … treats others with respect, even when opinions differ from their own. | |
| Overall |  |  | |
|  | This area of competence includes the handling, promotion and maintenance of professional competence, professional standards and attitude. Its core is to develop a professional identity. Reflection on one’s own competencies is an essential part of this skill. | | |

Quarterly, clinical supervisors assess these three areas of competence on a 7-point Likert scale. Situations suitable for the assessment of these competencies are videos of physician-patient contact, behaviour of the resident when working in a group, such as giving and receiving feedback, reflection-skills, respect toward others, the level of dept of reflection in the group about their actions in practice, working with the personalised learning plan, learning approach, discussion of written reflection reports.

Based on: Bosveld HEP, Essers GTJM, Guerrieri R, Heygele-Hamming JMW, Jobse P, Peet PG van, Schleypen H, Slieker M, Vangangel E, Vermeulen MI. Competentie Beoordeling Lijst (ComBeL) Versie JAAR 3 opleidingsperiode huisarts. Landelijke overleggroep Toetsing Februari 2019. English translation by Godschalx JA, Sijbom CAM, Barnhoorn PC, Mook WNKA van, which was approved by Huisartsenopleiding Nederland.
